# Initial Cytotoxicity of Additively and Subtractively Manufactured Resin‐Based Materials for Interim Fixed Dental Prostheses: An In Vitro Study

**DOI:** 10.1002/cre2.70413

**Published:** 2026-07-16

**Authors:** José M. Alegre, Jesús Peláez, Aránzazu Sánchez, Blanca Herrera, Seyed Ali Mosaddad, Pedro Diaz, María J. Suárez

**Affiliations:** ^1^ Department of Conservative Dentistry and Prosthodontics, Faculty of Dentistry Complutense University of Madrid Madrid Spain; ^2^ Department of Biochemistry and Molecular Biology, Faculty of Pharmacy Complutense University of Madrid Madrid Spain; ^3^ Health Research Institute of the “Hospital Clínico San Carlos” (IdISSC) Madrid Spain; ^4^ Biomedical Research Networking Center in Hepatic and Digestive Diseases (CIBEREHD‐ISCIII) Madrid Spain; ^5^ Department of Research Analytics, Saveetha Dental College and Hospitals, Saveetha Institute of Medical and Technical Sciences Saveetha University Chennai India; ^6^ Department of Prosthodontics, School of Dentistry Shiraz University of Medical Sciences Shiraz Iran

**Keywords:** cell survival, cytotoxicity, dental materials, fibroblasts, three‐dimensional printing

## Abstract

**Objectives:**

To compare the initial cytotoxic response of additively manufactured (AM) and subtractively manufactured (SM) resin‐based materials for interim fixed dental prostheses (FDPs) under indirect and direct in vitro exposure conditions.

**Material and Methods:**

A total of 216 standardized resin discs were fabricated from AM resins (Group P, *n* = 108) and SM polymethyl methacrylate (PMMA)‐based resins (Group M, *n* = 108). L929 mouse fibroblasts were used as the biological model, with a negative control group (Group C, *n* = 36). After 24 h of cell attachment, cell viability was assessed after 72 h under two conditions: indirect exposure to resin eluates and direct contact with resin discs. Viability was quantified by crystal violet staining. Data were analyzed using Kruskal–Wallis tests followed by Dunn's pairwise post hoc comparisons (*α* = 0.05).

**Results:**

In the indirect assay, Group M showed 87% cell survival relative to the control, whereas Group P showed 65%; in the direct assay, values were 89% and 48%, respectively. Significant differences were observed among pooled groups in both assays (*p* < 0.001). Based on ISO 10993‐5 thresholds, Group P was classified as slightly cytotoxic in the indirect assay and moderately cytotoxic in the direct assay, whereas Group M remained within the non‐cytotoxic to slightly cytotoxic range. Group P showed significantly lower cell viability than Group M in both assays (adjusted *p* < 0.001), while no statistically significant differences were detected within each manufacturing category (adjusted *p* > 0.05).

**Conclusions:**

At 72 h, the tested AM resins showed significantly lower cell viability than the tested SM PMMA‐based resins under the present in vitro conditions.

**Clinical Significance:**

The lower cell viability observed for the tested 3D‐printed interim materials under short‐term in vitro conditions highlights the need for caution when extrapolating 72‐h cell‐viability findings to clinical performance and supports further standardized long‐term in vitro and in vivo investigations.

## 1 Introduction

Additively manufactured (AM) resin‐based materials are increasingly used for the fabrication of interim fixed dental prostheses (FDPs) because they enable rapid, reproducible, and digitally controlled production. These materials have been proposed as alternatives to conventional self‐polymerizing resins and subtractively manufactured (SM) polymethyl methacrylate (PMMA)‐based CAD/CAM blocks, which are commonly used for interim restorations. Compared with conventional provisional materials, digitally fabricated resin‐based materials may offer improved workflow efficiency, dimensional consistency, and clinical adaptability for tooth‐ or implant‐supported interim FDPs (Atria et al. 2022; Guerrero‐Gironés et al. 2022; Jain et al. 2022; Park et al. 2020; Raszewski et al. 2022; Saini et al. 2024; Scotti et al. 2020).

Despite these advantages, the biological safety of AM resin‐based materials remains a relevant concern. Printable resins are generally light‐polymerized materials whose cytocompatibility may be influenced by resin formulation, degree of monomer conversion, polymer network structure, filler content, and photoinitiator system. Incomplete polymerization or insufficient post‐processing may lead to the release of residual monomers, photoinitiators, and degradation products, collectively referred to as resin eluates. These substances may diffuse into saliva and surrounding oral tissues and may induce local cytotoxic or inflammatory responses (Alshamrani et al. 2022; Atria et al. 2022; Aydın et al. 2023; Folwaczny et al. 2023; Frasheri et al. 2022; Kim et al. 2022; Park et al. 2020; Revilla‐León et al. 2019; Sa et al. 2019). Salivary esterases may further contribute to the degradation of resin components and facilitate the release or uptake of biologically active products by oral tissues (Çakmak et al. 2024; Hwangbo et al. 2021; Lin et al. 2020; Namsoy et al. 2024; Tahayeri et al. 2018).

Current evidence indicates that cytotoxic behavior is not determined by manufacturing category alone. Rather, material‐specific composition, polymerization history, and post‐processing conditions appear to play important roles in the biological response of resin‐based materials (Alshamrani et al. 2022; Aydın et al. 2023; Bürgers et al. 2022; Folwaczny et al. 2023; Hasanzade et al. 2023; Mudhaffer et al. 2025; Turkalj et al. 2025; Wedekind et al. 2021). For AM resins, washing duration, cleaning solution, post‐polymerization protocol, and oxygen‐inhibition control may affect residual monomer release and cell viability (Atria et al. 2022; Jin et al. 2022; Nam et al. 2023). In contrast, SM PMMA‐based blocks are industrially polymerized, often under controlled high‐temperature and high‐pressure conditions, which may reduce residual monomer content and improve polymer network stability.

In vitro cytotoxicity studies have commonly evaluated dental resin‐based materials using fibroblast cell lines, including human gingival fibroblasts and L929 mouse fibroblasts, and viability assays such as MTT, XTT, WST‐based assays, and crystal violet staining (Aydın et al. 2023; Folwaczny et al. 2023; Revilla‐León et al. 2019). Although several studies have compared AM resins with pre‐polymerized SM PMMA‐based or conventionally processed resin materials, differences in experimental protocols, post‐processing methods, exposure conditions, biological models, and outcome assessment methods make direct comparison across studies difficult. In addition, fewer investigations have assessed indirect exposure and direct material–cell contact within the same standardized experimental framework for resin‐based materials intended for interim FDPs.

Therefore, the present study aimed to assess and compare the initial cell viability response of resin‐based materials fabricated by additive manufacturing with that of SM PMMA‐based resins used for interim FDPs under standardized indirect exposure and direct contact conditions. The null hypothesis was that no significant differences in cell viability would be observed—under either indirect exposure or direct contact conditions—between the tested additively and SM resin‐based materials.

## 2 Materials and Methods

### 2.1 Specimen Design and Material Grouping

**Figure 1:**
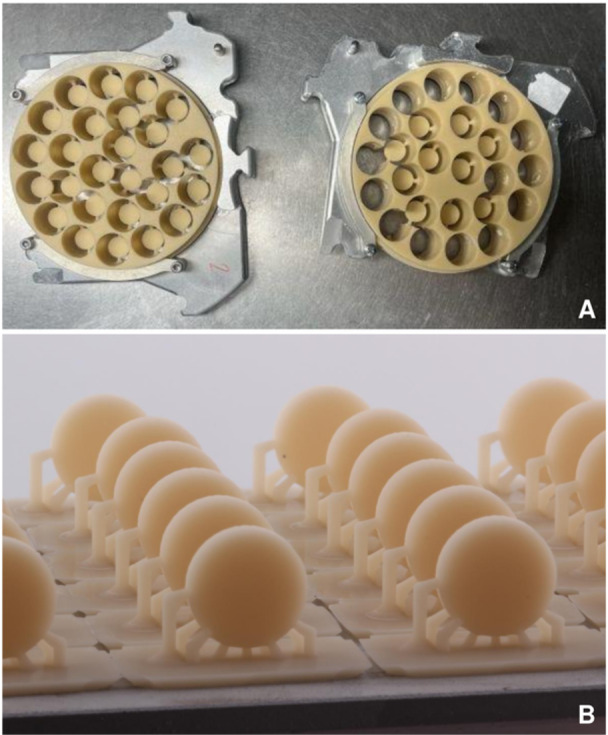
Representative specimens used in the study: (A) subtractively manufactured PMMA resin discs (IDODENTINE, IDO) and (B) additively manufactured resin‐based discs produced by 3D printing (GC Temp Print, GCTP).

A total of 216 resin discs were designed using Exocad Dental CAD software (Exocad GmbH, Darmstadt, Germany). Each disc had a diameter of 10 mm and a thickness of 2 mm, consistent with dimensions recommended for in vitro cytotoxicity testing (Turkalj et al. 2025). Specimens were divided into two experimental groups (*n* = 108 per group): SM PMMA resin discs (Group M) and AM resin‐based discs fabricated by 3D printing (Group P). Group M consisted of three CAD/CAM‐milled PMMA resins—IDODENTINE (IDO), 4 DESIGN 4 DISKS (DES), and Telio CAD (TEL)—whereas Group P included three printable resin‐based materials: GC Temp Print (GCTP), P Pro Crown & Bridge (PCB), and VarseoSmile Temp (VSM) (Figure 1). The composition and manufacturing characteristics of all materials are summarized in Table 1.

**Table 1:** Characteristics of the resin‐based materials used in the study.

Manufacturing method	Material (group)	Brand/manufacturer	Batch	Material type	Manufacturing technique	Composition		Polymerization
						Filler content (wt%)	Polymer matrix composition (wt%)	
Additive manufacturing (3D‐printed resin)	GC Temp Print (GCTP)	GC Europe A.G., Leuven, Belgium	2008071	Methacrylic acid ester‐based resin	Additive	10% to < 25% quartz	≥ 50% to < 75% UDMA; 10% to < 25% TEGDMA; 2.5%–5% EDMA, ethoxylated bisphenol‐A derivatives, 2‐methylprop‐2‐enoic acid	Light‐polymerized (TPO < 2.5%)
	P Pro Crown & Bridge (PCB)	Straumann, Basel, Switzerland	210514A	Methacrylic acid ester‐based resin	Additive	NR	20%–40% acrylic resin; < 25% UDMA; < 10% acrylic resin; < 1% TMPTA	Light‐polymerized (TPO < 1%)
	VarseoSmile Temp (VSM)	BEGO, Bremen, Germany	600324	Methacrylic acid ester‐based resin	Additive	30%–50% silanized dental glass	≥ 50% to < 75% Bis‐EMA	Light‐polymerized (TPO < 2.5%, MBF)
Subtractive manufacturing (CAD/CAM block)	4 DESIGN 4 DISKS (DES)	Shandong Huge Dental Material Corp., Shandong, China	250620010	PMMA	Subtractive	< 1% mineral pigments	> 99% PMMA; dibenzoyl peroxide	HT‐HP
	IDODENTINE (IDO)	Unión Dental S.A. (UNIDESA‐ODI), Tielmes, Madrid, Spain	J20	PMMA	Subtractive	< 1% mineral pigments and non‐toxic additives	> 99% PMMA; dibenzoyl peroxide	HT‐HP
	Telio CAD (TEL)	Ivoclar Vivadent S.L., Schaan, Liechtenstein	Z0179Y	PMMA	Subtractive	< 1% pigments	99.5% PMMA	HT‐HP

Abbreviations: Bis‐EMA, bisphenol‐A ethoxylated dimethacrylate; EDMA, ethylene dimethacrylate; HT‐HP, high temperature–high pressure; MBF, methyl benzoylformate; NR, not reported; PMMA, polymethylmethacrylate; TEGDMA, triethylene glycol dimethacrylate; TMPTA, trimethylolpropane triacrylate; TPO, 2,4,6‐trimethylbenzoyl phosphine oxide; UDMA, urethane dimethacrylate.

### 2.2 Sample Size Calculation and Experimental Unit

Sample size calculation was performed using G*Power software (version 3.1.9.7; Heinrich Heine University Düsseldorf, Düsseldorf, Germany) and was based on previously published in vitro cytotoxicity studies evaluating resin‐based dental materials (Aydın et al. 2023; Guerrero‐Gironés et al. 2022; Lin et al. 2020; Wuersching et al. 2022). Power analysis was performed using an alpha level of 0.05, a statistical power of 0.80, and an effect size of 0.25. The selected effect size was considered appropriate for detecting moderate differences in cell viability among the study groups based on the magnitude of differences reported in comparable in vitro studies. This yielded a required sample size of 54 discs per manufacturing approach per assay. Accordingly, for each assay (Trial 1: indirect exposure; Trial 2: direct contact), 18 discs per material were tested (*n* = 18). As six materials were evaluated, including three SM PMMA resins and three AM printable resins, this corresponded to 108 resin discs per assay (6 materials × 18 discs). Therefore, across both assays, a total of 216 resin discs were analyzed. The experimental unit used for statistical analysis was a single resin disc, with one disc considered one independent observation. When technical optical‐density readings were obtained from the same well or experimental unit, these values were averaged to generate a single final value for that disc before statistical analysis. Technical readings from the same disc were not entered separately into the statistical model.

### 2.3 Specimen Fabrication and Post‐Processing

AM specimens were produced using Chitubox Dental software (Shenzhen, China) and an SLA 3D printer with a 35 µm resolution and a 405 nm LED matrix 2.0 light source (Phrozen Sonic Mini 4K, Phrozen Technology, Hsinchu City, Taiwan). All specimens were printed in a vertical (90°) build orientation and a 30 µm‐layer thickness, which were kept constant for all printable materials to minimize variability related to printing parameters. The printer resolution was 3840 pixels along the *X*‐axis and 2160 pixels along the *Y*‐axis. Following fabrication, printed discs were released from the build platform using a spatula and underwent a standardized post‐processing protocol. Specimens were cleaned in two sequential steps using 96% ethanol in an unheated ultrasonic bath: first, discs were ultrasonically cleaned for 3 min in a reusable ethanol solution, followed by a second ultrasonic cleaning for 2 min in a fresh 96% ethanol bath. After ultrasonic cleaning, specimens were removed from the bath, and resin remnants were gently removed using a brush soaked in ethanol. Specimens were then post‐polymerized using a high‐intensity xenon‐flash light‐polymerization unit (Otoflash G171; NK‐Optik, Baierbrunn, Germany) using 2000 flashes per side (top and bottom; Σ 4000 flashes). To reduce oxygen inhibition and minimize the formation of an inhibition layer during curing, discs were placed in a transparent container filled with glycerin and photo‐polymerized under the glycerin barrier for 10 min; specimens were then rinsed to remove residual glycerin. A single post‐polymerization protocol was applied to all AM specimens as a standardization strategy to minimize variability related to post‐processing conditions and allow direct comparison among the tested printable materials.

Finally, discs were ultrasonically rinsed in distilled water for 2 min, air‐blown with compressed air, and allowed to dry for 30 min in a drying cabinet at approximately 37°C before cytotoxicity testing. Subtractive specimens were dry milled using a five‐axis milling unit (Zenotec Select Hybrid, Ivoclar Vivadent, Schaan, Liechtenstein). Following fabrication, all discs were sequentially polished using silicon carbide abrasive pads designed for acrylic materials (coarse, medium, and fine grit) at rotational speeds of 5000–7000 rpm, following the manufacturer's recommendations. After polishing, all specimens from both manufacturing groups were ultrasonically cleaned in distilled water for 5 min to remove surface debris, then thoroughly rinsed with distilled water, air‐dried with oil‐free compressed air, and stored individually in sterile, sealed containers (handled with clean nitrile gloves and sterile tweezers) until cytotoxicity testing.

No additional terminal sterilization procedure was performed after the standardized cleaning, rinsing, and drying protocol, to avoid potential alteration of the resin surface or residual chemical profile before cytotoxicity testing. After cleaning, specimens were handled using sterile tweezers, stored individually in sterile sealed containers, and introduced into the cell culture workflow inside a Class II biological safety cabinet under aseptic conditions.

### 2.4 Cytotoxicity Testing Conditions and Cell Culture

Cytotoxicity testing was conducted at the Department of Biochemistry and Molecular Biology, Faculty of Pharmacy, Complutense University of Madrid. The experimental procedures were performed with reference to ISO 10993–5:2009 (International Organization for Standardization 2009) and ISO 10993–12:2021 (International Organization for Standardization 2021), including the use of an established fibroblast cell line, extract‐based and direct‐contact exposure conditions, and standardized sample preparation procedures. However, a positive control reference material was not included; therefore, confirmation of assay sensitivity should be considered a methodological limitation. Mouse fibroblast L929 cells (lot no. 85011425; Sigma‐Aldrich, St. Louis, MO, USA) were used as the biological model. Cells were cultured under standard conditions (37°C, 5% CO_2_) in Dulbecco's Modified Eagle's Medium (DMEM; Gibco/Life Technologies, Carlsbad, CA, USA) supplemented with 10% fetal bovine serum (FBS; Merck, Darmstadt, Germany). A negative control group (Group C) consisted of cells cultured without exposure to the material and included 18 independent wells per assay (*n* = 18), for a total of *n* = 36 across both assays.

### 2.5 Indirect Exposure and Direct Contact Assays

**Figure 2:**
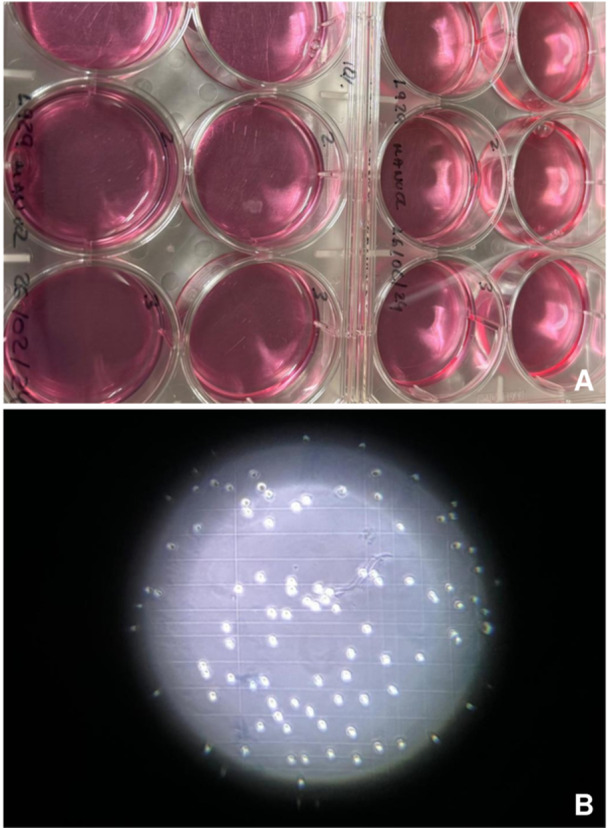
L929 mouse fibroblast cells used for cytotoxicity assessment: (A) cells cultured under standard conditions in growth medium and (B) microscopic view of L929 cells during routine observation.

Preliminary experiments identified a seeding density of 2.5 × 10^4^ cells per well in six‐well plates under serum‐free conditions as optimal for cytotoxicity assessment. Cells were thawed, expanded to approximately 80% confluence, and routinely passaged by trypsinization. Cell counts were determined using a Neubauer hemocytometer following Trypan Blue staining to distinguish viable from non‐viable cells (Mudhaffer et al. 2025) (Figure 2). After seeding, cells were allowed to attach for 24 h in DMEM containing 10% FBS, after which the medium was replaced with serum‐free DMEM. Following an equilibration period of 1–2 h, cytotoxicity testing was initiated.

**Figure 3:**
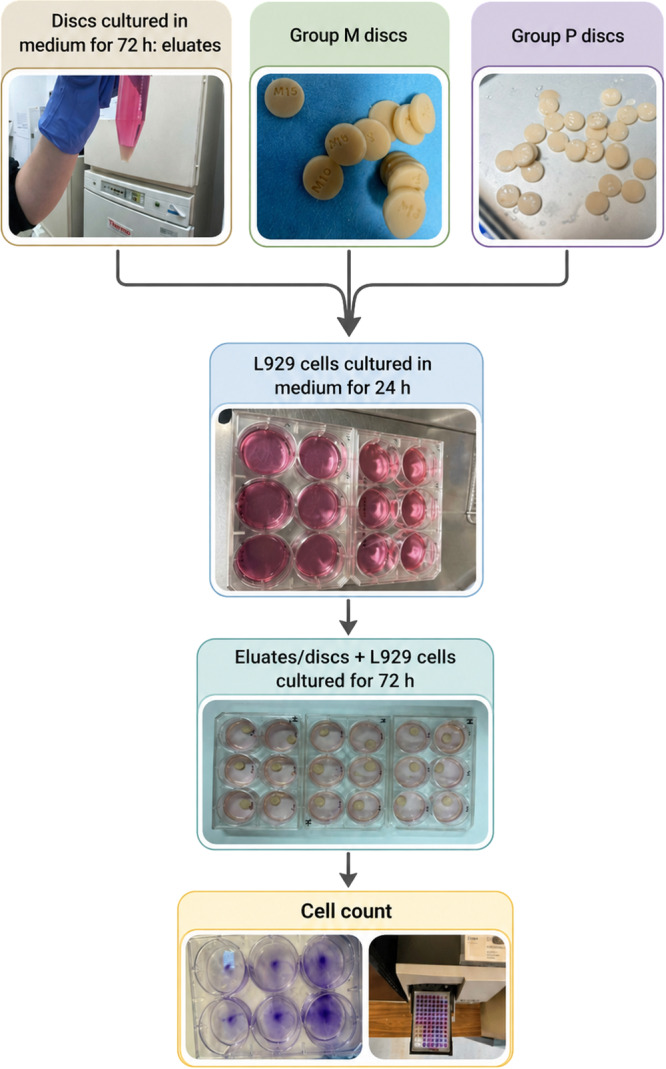
Schematic workflow of the experimental protocol illustrating specimen fabrication, cell culture conditions, indirect exposure and direct contact assays, and quantitative cell viability analysis.

Two experimental assays were performed. In Trial 1 (indirect exposure assay), resin discs were preincubated in serum‐free DMEM (Gibco/Life Technologies, Carlsbad, CA, USA) for 72 h to generate eluates. The resulting extracts were filtered through a 0.22 μm pore‐size membrane and subsequently applied to cultured cells. In parallel, a blank negative‐control extract was prepared by incubating serum‐free DMEM for 72 h under identical conditions and filtering it in the same manner, without material exposure, and this medium was applied to the negative control wells. In Trial 2 (direct contact assay), resin discs were placed directly in contact with the cell monolayer (Figure 3). For all experimental conditions, L929 cells were initially allowed to attach for 24 h in DMEM supplemented with 10% FBS (Merck, Darmstadt, Germany), after which the medium was replaced with serum‐free DMEM. Following an equilibration period of 1–2 h, cytotoxicity testing was initiated, and cells were incubated under standard culture conditions (37°C, 5% CO_2_) for 72 h, with no further medium change during the exposure period. The negative control was subjected to the same medium composition, serum conditions, medium‐change protocol, and 72‐h incubation period as the experimental groups. All procedures were carried out inside a Class II biological safety cabinet (Telstar BioUltra, Terrasa, Barcelona, Spain) to maintain a sterile environment during cell handling.

### 2.6 Cell Viability Assessment

Cell viability was quantified using crystal violet staining as previously described (Sánchez et al. 1996). Crystal violet staining was selected because it allows quantification of the adherent cell population remaining after material exposure and is suitable for comparing cell retention after indirect exposure and direct material–cell contact. Therefore, in the present study, cell viability was estimated based on adherent cell biomass rather than mitochondrial metabolic activity.

Briefly, cells were washed with phosphate‐buffered saline (PBS), stained with 1 mL of crystal violet solution per well for 20 min under gentle agitation (FinePcr Rocker, Finemould Precision, Dongguan City, China), rinsed thoroughly with water, and air‐dried. The dye was then solubilized by adding 1 mL of 1% sodium dodecyl sulfate (SDS) to each well and incubating for 20 min with agitation. Aliquots (200 μL) from each well were transferred to 96‐well plates, and optical density was measured at 570 nm using a microplate reader (Biotek PowerWave XS, Marshall Scientific, Hampton, NH, USA) (Figure 3). Blank wells containing crystal violet/SDS solution without cells were included, and their mean absorbance was subtracted from all experimental readings before calculating cell viability. Aliquots from each experimental condition were transferred to the 96‐well plates using a predefined plate layout to avoid systematic positioning of a single group in one plate region and to minimize potential plate‐position or edge‐related effects. Cell viability was expressed as a percentage relative to the negative control, as reported in comparable studies (Aydın et al. 2023; Guerrero‐Gironés et al. 2022; Hu et al. 2025; Wuersching et al. 2022). According to the International Organization for Standardization, 10993–5:2009, materials were classified as non‐cytotoxic (> 90% cell survival), slightly cytotoxic (60%–90%), moderately cytotoxic (30%–60%), or severely cytotoxic (< 30%). Cell viability below 70% was considered indicative of cytotoxicity.

### 2.7 Statistical Analysis

Normality of the data was assessed using the Kolmogorov–Smirnov test, and homogeneity of variances among groups was evaluated using the Brown–Forsythe test. Because the assumption of normal distribution was not satisfied, inferential comparisons among study groups were performed using the nonparametric Kruskal–Wallis test followed by Dunn's pairwise post hoc comparisons. Adjusted *p*‐values from Dunn's test were used for pairwise interpretation. Descriptive data are presented as mean ± standard deviation to facilitate comparison with previous cytotoxicity studies and as median with interquartile range (IQR) to support interpretation of the nonparametric analysis. All analyses were performed in GraphPad Prism (version 9.5.1), and *p*‐values less than 0.05 were considered statistically significant.

## 3 Results

**Figure 4:**
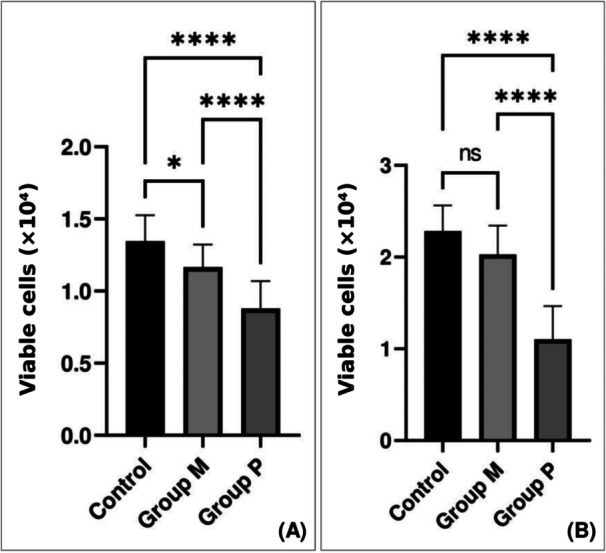
Bar plots showing mean ± SD viable‐cell counts (×10^4^ cells) for (A) the indirect exposure assay and (B) the direct contact assay based on pooled comparisons. Group P represents additively manufactured (3D‐printed) resins, and Group M represents subtractively manufactured (milled) polymethyl methacrylate (PMMA)‐based resins. ns indicates no statistically significant difference. Asterisks indicate statistically significant pairwise differences according to Dunn's post hoc test following the Kruskal–Wallis test: *adjusted *p* < 0.05; ****adjusted *p* < 0.0001.

Tables 2 and 3 summarize the cell count data obtained from the indirect exposure (Trial 1) and direct contact (Trial 2) assays, respectively. In Trial 1, Group M exhibited a mean cell survival of 87% relative to the control group (1.171 × 10^4^ vs. 1.345 × 10^4^ cells), corresponding to a 13% reduction in cell viability. In contrast, Group P showed a mean cell survival of 65% (0.879 × 10^4^ cells), corresponding to a 35% reduction in cell viability relative to the control group (Figure 4A).

**Table 2:** Trial 1 (Indirect exposure assay): Cell counts (×10^4^ cells).

Disc	Control (C)	Group M			Group P		
		DES	IDO	TEL	GCTP	PCB	VSM
1	1.329	1.218	1.261	1.241	0.616	0.696	0.566
2	1.345	1.097	1.097	1.099	0.763	0.764	0.683
3	1.273	0.940	0.940	0.931	1.037	1.037	1.036
4	0.891	0.901	0.801	0.901	0.847	0.846	0.867
5	1.189	1.151	1.149	1.172	0.718	0.798	0.798
6	1.238	1.112	1.114	1.108	0.776	0.777	0.778
7	1.251	1.207	1.207	1.239	0.721	0.723	0.721
8	1.412	1.243	1.242	1.245	0.933	0.931	0.953
9	1.207	1.101	1.100	1.153	0.751	0.754	0.751
10	1.367	1.314	1.110	1.318	1.012	1.079	1.182
11	1.577	1.626	1.624	1.618	0.969	0.990	0.970
12	1.353	1.051	1.049	1.071	0.793	0.791	0.793
13	1.457	1.157	1.102	1.139	0.599	0.598	0.597
14	1.352	1.131	1.130	1.183	1.026	1.025	1.046
15	1.753	1.314	1.312	1.318	1.383	1.385	1.383
16	1.322	1.203	1.201	1.215	1.031	1.031	1.031
17	1.544	1.102	1.157	1.144	0.818	0.881	0.818
18	1.356	1.171	1.097	1.141	0.819	0.898	0.899
Mean ± SD	1.345 ± 0.18	1.169 ± 0.16	1.165 ± 0.15	1.179 ± 0.17	0.867 ± 0.18	0.888 ± 0.17	0.881 ± 0.18
Median (IQR)	1.348 (1.256–1.401)	1.154 (1.101–1.215)	1.122 (1.098–1.206)	1.162 (1.116–1.241)	0.818 (0.754–1.001)	0.863 (0.767–1.016)	0.842 (0.758–1.016)
Total mean ± SD	1.345 ± 0.18^a^	1.171 ± 0.16^b^			0.879 ± 0.18ᶜ		
Total median (IQR)	1.348 (1.256–1.401)^a^	1.150 (1.100–1.234)^b^			0.832 (0.756–1.022)ᶜ		

*Note:* Values for individual discs represent the averaged cell‐count value obtained for each independent resin disc and are expressed as ×10^4^ cells. The mean ± SD and median (IQR) rows represent descriptive values calculated across the 18 independent discs within each material. The total mean ± SD and total median (IQR) rows represent pooled values for the control group (C), milled resins (M: DES, IDO, and TEL), and printed resins (P: GCTP, PCB, and VSM). Technical readings obtained from the same well/disc, when applicable, were averaged before statistical analysis and were not treated as independent observations. Different superscript letters indicate statistically significant differences according to Dunn's pairwise post hoc comparisons following the Kruskal–Wallis test (adjusted *p* < 0.05).

Abbreviations: C, control group; DES, 4 DESIGN 4 DISKS; GCTP, GC Temp Print; IDO, IDODENTINE; IQR, interquartile range; M, milled resins; P, printed resins; PCB, P Pro Crown & Bridge; SD, standard deviation; TEL, Telio CAD; VSM, VarseoSmile Temp.

**Table 3:** Trial 2 (direct contact assay): Number of viable cells (×10^4^).

Disc	Control (C)	Group M			Group P		
		DES	IDO	TEL	GCTP	PCB	VSM
1	2.492	2.141	1.661	2.841	1.007	0.696	0.566
2	2.435	2.053	2.097	1.499	1.302	0.764	1.623
3	2.491	1.917	1.740	1.971	0.857	1.737	1.036
4	2.026	2.077	1.601	1.991	0.988	0.846	0.867
5	2.064	1.651	1.549	1.972	0.958	0.798	0.778
6	1.953	1.780	1.514	1.808	0.878	1.977	1.748
7	2.088	2.001	1.917	2.239	0.913	0.763	0.721
8	2.293	2.253	1.842	1.945	1.101	0.931	0.953
9	2.279	2.077	1.910	1.953	0.655	1.974	1.711
10	2.323	2.147	2.910	2.318	1.049	1.079	1.182
11	2.582	2.199	1.624	1.818	1.111	0.990	0.970
12	2.237	2.260	2.049	2.971	1.123	0.891	0.793
13	2.064	1.707	2.102	1.939	0.962	1.598	1.557
14	2.178	1.925	2.170	2.183	0.926	1.125	1.046
15	2.439	2.073	1.912	1.818	1.096	1.385	0.783
16	2.503	2.769	2.201	2.215	1.350	1.031	1.031
17	1.730	1.971	1.929	2.144	0.944	0.881	1.818
18	2.936	1.922	2.097	2.141	1.977	0.898	0.859
Mean ± SD	2.284 ± 0.280	2.051 ± 0.249	1.935 ± 0.328	2.098 ± 0.353	1.067 ± 0.278	1.131 ± 0.418	1.113 ± 0.397
Median (IQR)	2.286 (2.070–2.478)	2.063 (1.923–2.146)	1.915 (1.681–2.097)	1.982 (1.941–2.207)	0.998 (0.931–1.109)	0.961 (0.855–1.320)	1.001 (0.810–1.463)
Total mean ± SD	2.284 ± 0.280^a^	2.028 ± 0.315^a^			1.104 ± 0.364^b^		
Total median (IQR)	2.286 (2.070–2.478)^a^	1.982 (1.859–2.146)^a^			0.989 (0.870–1.168)^b^		

*Note:* Values for individual discs represent the averaged viable‐cell‐count value obtained for each independent resin disc and are expressed as ×10^4^ cells. The mean ± SD and median (IQR) rows represent descriptive values calculated across the 18 independent discs within each material. The total mean ± SD and total median (IQR) rows represent pooled values for the control group (C), milled resins (M: DES, IDO, and TEL), and printed resins (P: GCTP, PCB, and VSM). Technical readings obtained from the same well/disc, when applicable, were averaged before statistical analysis and were not treated as independent observations. Different superscript letters indicate statistically significant differences according to Dunn's pairwise post hoc comparisons following the Kruskal–Wallis test (adjusted *p* < 0.05).

Abbreviations: C, control group; DES, 4 DESIGN 4 DISKS; GCTP, GC Temp Print; IDO, IDODENTINE; IQR, interquartile range; M, milled resins; P, printed resins; PCB, P Pro Crown & Bridge; SD, standard deviation; TEL, Telio CAD; VSM, VarseoSmile Temp.

In Trial 2, Group M demonstrated a mean cell survival of 89% relative to the control group (2.028 × 10^4^ vs. 2.284 × 10^4^ cells), corresponding to an 11% reduction in cell viability. Conversely, Group P exhibited a marked decrease in mean cell survival to 48% (1.104 × 10^4^ cells), corresponding to a 52% reduction in cell viability relative to the control group (Figure 4B).

Based on ISO 10993‐5 cell‐survival thresholds, Group P was classified as slightly cytotoxic in the indirect exposure assay and moderately cytotoxic in the direct contact assay. In contrast, Group M remained within the non‐cytotoxic to slightly cytotoxic range under the tested conditions.

### 3.1 Pooled Comparisons

For the indirect exposure assay, the overall comparison among the three groups using the Kruskal–Wallis test indicated significant differences in cell viability (*p* < 0.001). Pairwise comparisons using Dunn's test revealed significant differences among all groups. The control group showed significantly higher cell viability than Group M (adjusted *p* = 0.021) and Group P (adjusted *p* < 0.001). Additionally, Group M exhibited significantly higher cell viability than Group P (adjusted *p* < 0.001). Overall, the highest cell viability values were observed in the control group, followed by Group M, whereas Group P demonstrated the lowest values, indicating the greatest cytotoxic effect among the pooled groups (Figure 4A).

For the direct contact assay, Dunn's multiple comparisons test revealed no significant difference in cell viability between the control group and Group M (adjusted *p* = 0.1014). However, both the control group and Group M showed significantly higher cell viability than Group P (adjusted *p* < 0.0001 for both comparisons). Overall, the control and Group M demonstrated comparable cell viability values, and both were significantly higher than those observed in Group P, indicating a greater cytotoxic effect for Group P under direct contact conditions (Figure 4B).

### 3.2 Material‐Specific Comparisons

**Figure 5:**
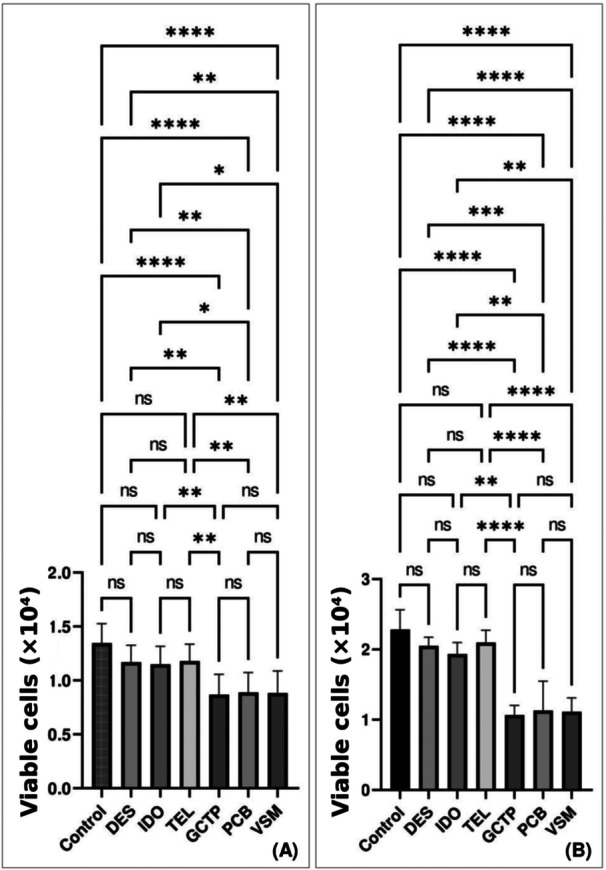
Bar plots showing mean ± SD viable‐cell counts (×10^4^ cells) for (A) the indirect exposure assay and (B) the direct contact assay based on material‐specific comparisons. The evaluated materials were GC Temp Print (GCTP), P Pro Crown & Bridge (PCB), VarseoSmile Temp (VSM), 4 DESIGN 4 DISKS (DES), IDODENTINE (IDO), and Telio CAD (TEL). ns indicates no statistically significant difference. Asterisks indicate statistically significant pairwise differences according to Dunn's post hoc test following the Kruskal–Wallis test: *adjusted *p* < 0.05; **adjusted *p* < 0.01; ***adjusted *p* < 0.001; ****adjusted *p* < 0.0001.

For the indirect exposure assay, the overall comparison among the seven groups using the Kruskal–Wallis test indicated significant differences in cell viability (*p* < 0.001), and therefore, Dunn's multiple comparisons test was performed. The results showed that the control group did not differ significantly from DES, IDO, or TEL (all adjusted *p* > 0.05), whereas the control group showed significantly higher cell viability than GCTP, PCB, and VSM (all adjusted *p* < 0.001). Similarly, the DES, IDO, and TEL groups showed significantly higher cell viability than GCTP, PCB, and VSM (adjusted *p* ranging from 0.001 to 0.020). No significant differences were found among DES, IDO, and TEL groups (all adjusted *p* > 0.999). In addition, no statistically significant differences were detected among GCTP, PCB, and VSM under the conditions tested (all adjusted *p* > 0.999). Overall, the findings indicate that the control, DES, IDO, and TEL groups exhibited comparable cell viability in the indirect assay, and all showed significantly higher viability than GCTP, PCB, and VSM, while the latter three groups showed reduced cell viability values, with no statistically significant differences detected among them under the conditions tested (Figure 5A).

For the direct contact assay, pairwise comparisons using Dunn's test showed no significant differences in cell viability between the control group and DES, IDO, and TEL groups (all adjusted *p* > 0.05). However, the control group demonstrated significantly higher cell viability than GCTP, PCB, and VSM (all adjusted *p* < 0.001). Similarly, DES and TEL showed significantly higher cell viability than GCTP, PCB, and VSM (adjusted *p* ≤ 0.001). The IDO group also exhibited significantly higher cell viability than GCTP, PCB, and VSM (adjusted *p*‐values ranging from 0.003 to 0.005). No statistically significant differences were observed among DES, IDO, and TEL groups (all adjusted *p* > 0.999). Furthermore, no statistically significant differences were detected among GCTP, PCB, and VSM under the conditions tested (all adjusted *p* > 0.999). Overall, the control, DES, IDO, and TEL groups showed comparable cell viability in the direct assay, and all showed significantly higher viability than GCTP, PCB, and VSM, while no statistically significant differences were detected within each subset under the conditions tested (Figure 5B).

## 4 Discussion

The findings of the present study demonstrated significant differences in cell viability between the tested Group P and Group M resin‐based materials under both indirect and direct exposure conditions, indicating differences in cytotoxic response and leading to rejection of the null hypothesis. In the indirect exposure assay, the tested SM PMMA‐based resins showed 87% mean cell survival relative to the control, whereas the tested AM resins showed 65% survival. In the direct contact assay, the corresponding values were 89% for the tested SM materials and 48% for the tested AM materials. Therefore, the reduction in cell viability associated with the tested AM materials was more pronounced under direct contact conditions than under eluate‐based exposure. Material‐specific comparisons did not detect statistically significant differences within either the SM or AM subgroups under the conditions tested. However, because manufacturing method and material composition are inherently confounded in the present study design, these findings should not be interpreted as isolating additive manufacturing itself as the primary determinant of cytotoxicity. Rather, the lower cell viability observed for the tested AM materials may reflect the combined influence of manufacturing approach, resin chemistry, polymer matrix, filler content, photoinitiator system, post‐processing conditions, and polymerization history.

When compared with previous investigations, the direction and magnitude of the present findings show both agreements and differences. Wuersching et al. (2022) reported lower initial biocompatibility for several printable resins used for FDPs compared with subtractively processed resin materials, which is consistent with the present finding that the tested AM materials showed lower cell viability than the tested SM PMMA‐based materials. The present study extends this comparison by showing that the difference was observed under both indirect exposure and direct contact conditions, with a greater reduction in viability in the direct contact assay. In contrast, Aydın et al. (2023) reported no statistically significant differences in cell growth between most 3D‐printed resins and resin‐based CAD/CAM blocks after 72 h, and cell viability values generally remained above the 70% cytotoxicity threshold. This contrasts with the present findings, in which the tested AM group showed 65% survival in the indirect exposure assay and 48% survival in the direct contact assay. Guerrero‐Gironés et al. (2022) also observed broadly comparable cytotoxic responses among most 3D‐printed and conventional resins, although one printable material showed more pronounced cytotoxicity, supporting the interpretation that biological response is highly material dependent. Similarly, Folwaczny et al. (2023) reported that 3D‐printed temporary restorative materials may affect cell viability in a material‐dependent manner when tested on human periodontal ligament cells. Taken together, these studies indicate that the cytotoxic response of printable and milled resin‐based materials cannot be attributed to manufacturing category alone, but should be interpreted in relation to material formulation, residual monomer release, photoinitiator content, post‐processing conditions, exposure model, and the cell type used for testing. Similarly, Şahin et al. (2025) compared temporary fixed prosthetic materials fabricated using conventional, CAD/CAM milling, and 3D‐printing methods and evaluated cytotoxicity in L929 mouse fibroblasts after extract exposure. The authors reported that the 3D‐printed material showed significantly greater cytotoxic effects than the conventional and CAD/CAM‐milled materials, whereas the CAD/CAM‐milled material exhibited high cell viability. These findings are consistent with the present results, in which the tested AM materials showed lower cell viability than the tested SM PMMA‐based materials under both indirect exposure and direct contact conditions. However, differences in extraction medium, exposure duration, material brands, and assay design should be considered when comparing the magnitude of the findings.

Various experimental models have been employed to assess the biocompatibility of dental materials, with animal studies and cell culture assays representing the most commonly used approaches in restorative dentistry (Murray et al. 2007). According to the International Organization for Standardization, 10993‐5:2009 (International Organization for Standardization 2009), in vitro cytotoxicity may be evaluated using direct contact, indirect contact with a physical barrier, or extract‐based methods. Lim et al. (2017) compared these approaches and reported superior sensitivity of the extract method when a single testing strategy is required. In the present investigation, both extract‐based (indirect) and direct contact assays were employed, enabling complementary assessment of cytotoxic responses. The extract method allowed evaluation of eluate‐mediated effects, whereas the direct contact assay simulated immediate material–cell interactions, providing a more comprehensive biological characterization. In the direct contact assay, surface‐related factors may also have influenced the measured adherent cell population independently of chemical cytotoxicity. Differences in surface roughness, surface energy, printing morphology, polishing characteristics, and oxygen‐inhibition layer management may affect fibroblast attachment, spreading, and retention on resin surfaces. Therefore, the lower cell viability observed under direct contact conditions may reflect the combined effects of material‐derived eluates and surface‐mediated differences in cell adhesion. Future studies incorporating surface roughness, wettability, surface topography, and chemical elution analyses would help distinguish chemical cytotoxicity from surface‐related effects on fibroblast adhesion.

The biological safety of resin‐based materials is essential for their clinical application, particularly for interim restorations that remain in prolonged contact with oral tissues. Resin materials may release unreacted monomers, photoinitiators, and degradation products following polymerization, collectively referred to as eluates, which can initially affect the oral mucosa and subsequently diffuse toward deeper tissues via saliva or gingival crevicular fluid (Rogers et al. 2021). Fibroblasts, selected in this study as representative connective‐tissue cells, play a crucial role in extracellular matrix synthesis, wound healing, immune modulation, and epithelialization. Sustained exposure to toxic resin components can compromise fibroblast viability and function, potentially delaying tissue repair and increasing susceptibility to periodontal inflammation (Folwaczny et al. 2023; Guerrero‐Gironés et al. 2022; Hwangbo et al. 2021; Park et al. 2020; Wuersching et al. 2022).

The observed differences in cell viability may be associated with variations in resin composition, initiator chemistry, polymerization history, and post‐processing conditions (Mosaddad 2025). Although conventional milled resins and printable resins may share some methacrylate‐based components, including urethane dimethacrylate (UDMA) and triethylene glycol dimethacrylate (TEGDMA), their formulations can differ substantially in viscosity, filler content, initiator system, and polymer network structure. In the present study, the tested SM PMMA‐based materials were industrially polymerized and included thermal initiator systems, such as dibenzoyl peroxide, whereas the tested printable materials contained photoinitiator systems such as diphenyl (2,4,6‐trimethylbenzoyl) phosphine oxide (TPO) and/or methyl benzoylformate (MBF). Therefore, the relevant distinction concerns initiator chemistry and polymerization pathway rather than initiator absence. Printable resins generally require lower viscosity to ensure adequate flow and printability, which may be achieved through specific monomer combinations and diluent components (Atria et al. 2022; Kessler et al. 2020; Taormina et al. 2018). These formulation and processing differences may contribute to differences in eluate release and cellular response (Aydın et al. 2023); however, this interpretation remains hypothetical because residual monomer release, degree of conversion, and individual eluate profiles were not directly measured in the present study.

Post‐polymerization procedures may improve monomer conversion in printable resins; however, the effectiveness of these procedures depends on resin formulation, light exposure parameters, specimen geometry, oxygen inhibition, and post‐processing protocol. In contrast, SM PMMA blocks are industrially polymerized under controlled conditions, which may result in a more stable polymer network and lower residual monomer availability. These factors may partly explain the higher cell viability observed in Group M; however, this explanation should be interpreted cautiously because the present study did not directly quantify residual monomer content, degree of conversion, or the chemical composition of released eluates (Aydın et al. 2023; Lin et al. 2020; Wuersching et al. 2022).

The cytotoxic response observed in Group P cannot be attributed to a single compositional factor. Although manufacturer data indicate monomer contents below 75 wt%, this information alone is insufficient to fully explain the biological behavior. The use of undiluted eluates in the present study represents a worst case exposure scenario that does not fully replicate intraoral conditions, where continuous salivary dilution may mitigate toxic effects over time. Nevertheless, assessment of diluted eluates at multiple time points would be required to substantiate this assumption. Interpretation of the results is further limited by incomplete disclosure of material composition by manufacturers, particularly regarding filler content and specific monomer fractions, as exemplified by PCB resin. Initiator chemistry may also be relevant. The tested SM PMMA‐based materials were industrially polymerized and included thermal initiator systems, such as dibenzoyl peroxide, whereas the tested printable materials contained photoinitiator systems, including diphenyl TPO and/or MBF. TPO has been associated with cytotoxic and genotoxic effects in vitro (Michelsen et al. 2007; Popal et al. 2018; Van Landuyt et al. 2015; Wessels et al. 2015), and GCTP and VSM reportedly contain up to 2.5 wt% TPO. Therefore, photoinitiator‐related eluates may have contributed to the reduced cell viability observed in printable materials; however, this remains a proposed explanation because the chemical composition of released eluates was not directly analyzed in the present study. It is important to emphasize that the compositional information available for the materials investigated confirms compliance with CE Class IIa (1907/2006) certification, permitting intraoral use for up to 30 days (Aydın et al. 2023; Lin et al. 2020; Revilla‐León et al. 2019; Wuersching et al. 2022). Therefore, the present findings should be interpreted within the context of short‐term provisional applications.

Additional factors influencing cytocompatibility include printing technology and processing parameters, which may alter the physical and chemical properties of printed resins (Ide et al. 2017). Although higher cell viability has been reported for samples produced using DLP systems compared with SLA systems, these differences have not consistently reached statistical significance (Aydın et al. 2023). In the present study, all printable specimens were fabricated using a single SLA printer, with standardized preprocessing, washing, and post‐curing procedures. Incubation time has also been shown to affect cytotoxic responses, with increased toxicity observed after prolonged exposure periods (Aydın et al. 2023; Bayarsaikhan et al. 2021; Kim et al. 2022). Kim et al. (2022) reported that 3D‐printed crown and bridge resins exhibit increased cytotoxicity after 48 h of incubation compared to 24 h. This aligns with the findings of Bayarsaikhan et al. (2021), who reported that cytotoxicity rapidly increases with longer incubation times, from 24 to 48 and 72 h. Aydın et al. (2023) observed that printable resins caused greater cytotoxic effects in HGF‐1 cells after 72 h than after 24 h. Based on these findings, a 72‐h incubation period was selected to capture biologically relevant cytotoxic effects.

Clinical translation of these findings should be approached cautiously. The present in vitro model does not reproduce oral fluid dynamics, salivary dilution, esterase‐mediated degradation, acquired pellicle formation, or the continuous clearance of eluates that may occur intraorally. These factors may modify the concentration, diffusion, and biological activity of released resin components over time. Although the tested materials are indicated for temporary intraoral use under CE Class IIa certification, the present findings reflect short‐term cell viability responses under controlled laboratory conditions and should not be directly extrapolated to clinical performance. L929 fibroblasts were used because they are widely accepted for initial cytotoxicity screening; however, confirmation using human oral cell models, including gingival fibroblasts, periodontal ligament cells, and epithelial cells, would provide greater clinical relevance.

Several limitations must be acknowledged. The investigated materials represent only a limited selection of printable and milled resins, which restricts external validity. Material composition was not independently verified by spectroscopy or chromatography and relied on manufacturer‐reported data; therefore, residual monomer release, degree of conversion, and individual eluate profiles could not be confirmed. Although manufacturer‐recommended washing and post‐polymerization procedures were followed, incomplete removal of residual components cannot be excluded, and differences in post‐processing protocols across studies may limit comparability (Frasheri et al. 2022; Kim et al. 2022; Mosaddad 2025). Because a single standardized post‐polymerization protocol was applied to all AM materials, the biological outcomes should be interpreted as workflow‐specific and may differ from results obtained using manufacturer‐specific post‐polymerization protocols for each material. No separate terminal sterilization procedure was performed after cleaning, rinsing, and drying to avoid potential alteration of the resin surface or residual chemical profile; however, this should be considered a methodological limitation. To minimize contamination risk, specimens were handled with sterile instruments, stored individually in sterile containers, and introduced into the cell culture workflow inside a Class II biological safety cabinet. The findings therefore reflect initial biological interactions of freshly fabricated specimens rather than long‐term biocompatibility, as aging, water sorption, and mechanical degradation may influence monomer release over time (Aydın et al. 2023; Wuersching et al. 2022). In addition, the use of only L929 mouse fibroblasts limits biological extrapolation to the oral environment, where interim FDPs interact with multiple oral cell types, saliva, and gingival crevicular fluid. Furthermore, crystal violet staining estimates adherent cell biomass and is not directly equivalent to MTT, XTT, or WST‐based assays, which assess mitochondrial or metabolic activity. Therefore, comparisons with studies using metabolic viability assays should be interpreted cautiously, as assay‐specific differences may influence the magnitude of reported cell viability values. Finally, no positive control reference material was included; therefore, although group‐dependent differences were detected, assay sensitivity could not be fully verified.

Future investigations should include quantitative chemical characterization of eluates and correlation with biological outcomes using complementary analytical methods such as high‐performance liquid chromatography (HPLC), gas chromatography–mass spectrometry (GC–MS), Fourier‐transform infrared spectroscopy (FTIR), and Raman spectroscopy. These techniques would help quantify released monomers and photoinitiator‐related compounds, assess the degree of conversion, and validate whether residual monomer release, polymerization characteristics, or specific eluate profiles explain the observed differences in cell viability. Long‐term in vitro and in vivo studies employing standardized fabrication and post‐processing protocols are necessary to improve cross‐study comparability and clarify the clinical relevance of the initial cytotoxic responses observed in this study.

## 5 Conclusions

Within the limitations of this in vitro study, the following conclusions can be drawn:

1. Both additively and SM resin‐based materials exhibited measurable reductions in cell viability under the experimental conditions evaluated.
2. The tested AM (3D‐printed) resins demonstrated significantly lower cell viability than the tested SM (milled) PMMA‐based resins under both indirect (eluate‐based) and direct contact assays, whereas no statistically significant differences were detected among materials within each manufacturing category under the conditions tested.
3. The increased cytotoxic response observed for the tested 3D‐printed resins may be related to the combined influence of resin formulation, residual monomer release, photoinitiator content, post‐processing conditions, and polymerization characteristics compared with the tested industrially polymerized milled PMMA‐based resin blocks.
4. Standardization of fabrication and post‐processing protocols is essential to improve reproducibility and allow meaningful biological comparisons of resin‐based materials.
5. Further long‐term in vitro and in vivo studies incorporating a wider range of materials, human oral cell models, standardized post‐processing procedures, aging protocols, and more clinically representative exposure conditions are necessary to confirm these findings and better define their clinical relevance for interim FDPs.

## Author Contributions

**José M. Alegre:** conceptualization, formal analysis, writing – original draft preparation. **Jesús Peláez:** conceptualization, validation, writing – review and editing. **Aránzazu Sánchez:** conceptualization, methodology, data curation, writing – review and editing. **Blanca Herrera:** conceptualization, methodology, data curation, writing – review and editing. **Seyed Ali Mosaddad:** conceptualization, methodology, validation, writing – review and editing. **Pedro Diaz:** conceptualization, validation, data curation, and writing – review and editing. **María J. Suárez:** conceptualization, validation, writing – review and editing, supervision. All authors have read and agreed to the published version of the manuscript.

## Funding

The authors have nothing to report.

## Ethics Statement

The authors have nothing to report.

## Consent

The authors have nothing to report.

## Conflicts of Interest

The authors declare no conflicts of interest.

## Data Availability

The data that support the findings of this study are available from the corresponding author upon reasonable request.
